# Diversity of Silica-Scaled Chrysophytes in the Steppe Zone of the Southern Urals with a Description of a New Species from the Genus *Mallomonas*

**DOI:** 10.3390/life13112214

**Published:** 2023-11-16

**Authors:** Marina Ignatenko, Evgeniy Gusev, Tatyana Yatsenko-Stepanova

**Affiliations:** 1 Institute for Cellular and Intracellular Symbiosis of the Ural Branch of the Russian Academy of Sciences, 11 Pionerskaya Street, 460000 Orenburg, Russia; yacenkostn@gmail.com; 2 A.N. Severtsov Institute of Ecology and Evolution, Russian Academy of Sciences, 33 Leninsky Prospect, 119071 Moscow, Russia; evsergus@yahoo.com

**Keywords:** Southern Urals, silica-scaled chrysophytes, *Mallomonas*, *Synura*, stomatocyst

## Abstract

This paper is devoted to the study of the flora of silica-scaled chrysophytes in water bodies of the steppe zone of the Southern Urals (Russia). Twenty-four taxa were identified via scanning and transmission electron microscopy, twenty of which are representatives of the genus *Mallomonas* Perty, while four are species of the genus *Synura* Ehrenberg. In the course of the study, a species new to science from the genus *Mallomonas*, *M. baturinae* sp. nov., was described. This species belongs to the section *Papillosae*. The stomatocyst of *M. doignonii* was described. For the first time in Russia and for the third time since description, *M. phasma* and *M. solea-ferrea* var. *irregularis* were reported in the studied area. Here, their extended description is provided with illustrations of their scales in detail. Some rare taxa for the flora of Russia have been identified: *M. doignonii*, *M. pillula* f. *exannulata*, and *M. pillula* f. *valdiviana*. One taxon of the genus *Mallomonas* has not been identified to a species level and is probably a taxon new to science.

## 1. Introduction

Chrysophytes are a large group with more than 1200 described species in about 112 genera. They are widespread, predominantly freshwater planktonic protists, diverse in morphology [1]. Chrysophytes are unicellular or colonial algae characterized by heterokont flagella and chloroplasts with chlorophyll a and c, and by their endogenous silicified stomatocysts [1]. A special group of these consists of species whose cells are covered with siliceous structures (scales, bristles, spines). These organisms are represented in different phylogenetic lineages of chrysophytes from the orders Paraphysomonadales, Chromulinales, and Synurales [2]. The order Synurales is the most diverse, including more than 220 species of the genus *Mallomonas* [3,4] and more than 50 species of the genus *Synura* [5]. Identification of silica-scaled chrysophytes is based on the study of the ultrastructure of scales and bristles using scanning and transmission electron microscopy (SEM and TEM, respectively). For this group, a species concept based on the ultrastructural features of the silica structures is well-developed, which is in good agreement with molecular data [2,6,7]. This allows the use of chrysophyte algae with silica covers in paleoecological, biogeographical, and monitoring studies [8,9,10].

Silica-scaled chrysophytes of Russia have been studied very unevenly. The first studies of silica-scaled chrysophytes in Russia using electron microscopy began in the second half of the XXth century [11,12,13]. The flora of silica-scaled chrysophytes in Russia today remains insufficiently studied. Using the example of the genus *Mallomonas*, well studied throughout the world, in the territory of such a vast region as Russia, only 91 species and infraspecific taxa have been identified [12,13,14,15,16,17,18,19,20,21,22,23,24,25,26,27,28,29,30,31,32,33,34,35,36,37,38,39,40,41,42,43,44,45,46], although the climatic, geographical, and natural diversity of Russia suggests that biodiversity indicators may be significantly higher. For comparison, we can point out that in Europe, which is much smaller in area, 114 species and infraspecific taxa of the genus *Mallomonas* are known [47]. Almost every study of a new region in Russia leads to the discovery of rare species and taxa new to science or the flora of the country [36,39,40,41,42,43,44,48].

Initially, studies of silica-scaled chrysophytes primarily covered the territory of the central regions of the European part of Russia (reservoirs of the Volga basin) [11,12,13,22]. Until now, most of the work has been carried out in the central and northern regions of European Russia [20,21,22,23,24,25,26,27,28,29,49]. A number of interesting works have been published on the study of rivers, reservoirs, lakes, and swamps in Eastern Siberia, most of which have been carried out in the last decade [30,31,32,33,34,40]. The Ural region and Western Siberia have been studied to a much lesser extent [19,27,35,36,37,39]. However, studies of silica-scaled chrysophytes of the Polar Urals revealed a high diversity and abundant flora, including taxa new to science and to the flora of Russia [27,35,36]. The first data of silica-scaled chrysophytes of the Southern Urals, obtained using electron microscopy, are given in the studies of L.V. Snitko et al. [38,50,51,52,53,54]. However, these results cover only a part of this region excluding the steppe area.

It should be noted that the most important distinguishing feature of climate of the steppe is the limited precipitation. With a precipitation deficit, the importance of water bodies of different origin for the ecosystem increases. In the conditions of the steppe zone, they play a special role, forming the cores of biodiversity concentration [55,56,57].

The strong degradation of steppes as a result of anthropogenic human activity (ploughing, grazing, use of steppe reservoirs for irrigation), which is currently occurring, is the cause of the loss of steppe biodiversity in general, and the biodiversity of water ecosystems in particular [55,56,57]. In this situation, it is necessary to create protected natural areas for the conservation and study of biodiversity.

To date, the flora comprising the silica-scaled chrysophytes in water bodies of the protected natural areas of the Southern Urals, which are not subjected to serious anthropogenic pressure, remains completely unstudied. Numerous studies of chrysophytes from different regions, such as North America [58,59,60], Europe [61,62], Asia [63,64,65,66,67], and Russia [23,24,25,29], have shown that water bodies of the protected areas have a rich and distinctive flora and often include species new to science. The first studies of the protected natural areas of the Southern Urals identified 32 morphotypes of chrysophycean stomatocysts in the flora of Lake Zhurmankol, located on the territory of the Ashchisai Steppe site of the “Orenburgskii” State Nature Reserve, which indicates a wide variety of golden algae in this water body [68,69,70]. In addition, *Mallomonas rasilis* Dürrschmidt, a new species of chrysophytes for the flora of Russia, was also recorded in Lake Zhurmankol [14]. The data obtained served as a prerequisite for further research on the study of the diversity of the flora of chrysophytes in the natural protected areas of the Southern Urals.

The purpose of this work was to study the diversity of silica-scaled chrysophytes in water bodies of natural protected areas of the steppe zone of the Southern Urals using scanning and transmission electron microscopy.

## 2. Materials and Methods

**Figure 1:**
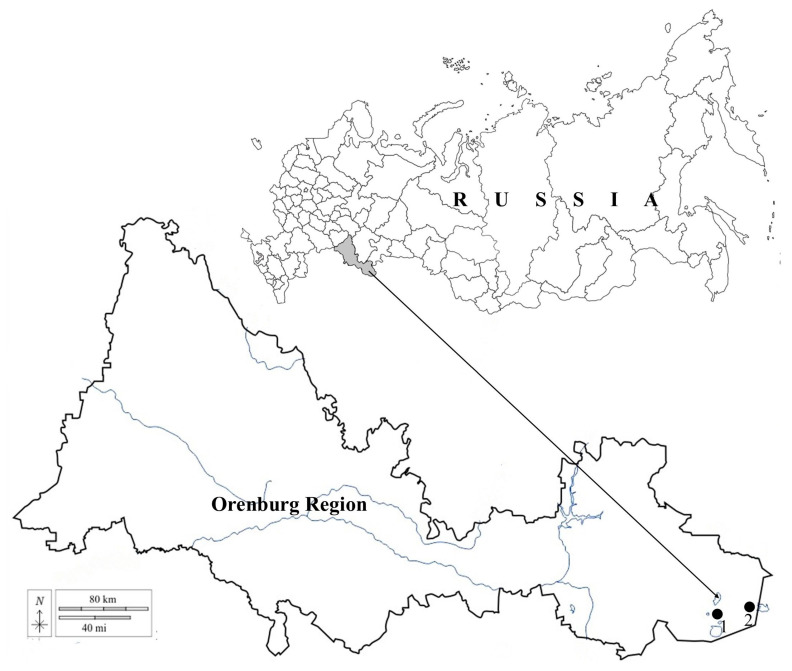
Schematic map of the study area: (**1**) “Svetlinskii” Biological Reserve, (**2**) “Orenburgskii” State Nature Reserve.

The material for the study comprised integrated samples (plankton, epipelon, and epilithon) taken during the open water period from four reservoirs located in the territory of the Aschisai Steppe site (“Orenburgskii” State Nature Reserve, Southern Urals, Russia) and the territory of the “Svetlinskii” Biological Reserve (Southern Urals, Russia) (Figure 1 and Table 1). These reservoirs are small and shallow. Their filling occurs mainly in the spring due to snowmelt; they are fed by rain, and there is no recharge by ground and spring waters [71]. Water replenishment varies greatly over the years and seasons, with some reservoirs completely drying up (lakes Nezametnoe, Zhurmankol, Liman).

Sampling was carried out in May 2020, April–May 2022, and May 2023. Sampling was carried out in the spring (April, May), since it is known that in temperate latitudes the greatest diversity of chrysophytes is observed in the spring [1,72,73]. In addition, the specified timing of sampling is determined by the temporary boundaries of the existence of the studied reservoirs–reservoirs are ephemeral, drying up quickly. Samples were fixed with a 40% formaldehyde solution and concentrated via the sedimentation method. Water temperature and pH were measured using a portable pH/°C analyzer HI98127 (Hanna Instruments, Inc., Woonsocket, RI, USA), and salinity was measured using an ANION 4100 laboratory analyzer (Infraspak-Analyte, Novosibirsk, Russia).

The study of the species composition of silica-scaled chrysophytes was carried out using scanning electron microscopy (SEM) on a Tescan Mira3 microscope (Tescan Brno, s.r.o, Brno, Czech Republic) at the Gagarin Center for the Identification and Support of Talented Children, (Orenburgskaya oblast) and also using transmission electron microscopy (TEM) on a JEM-1011 transmission electron microscope in the Center of Electron Microscopy at Papanin’s Institute for Biology of Inland Waters, RAS. An aliquot of a sample was washed from the fixative with distilled water via repeated centrifugation (5 min at 3000 rpm) using a Microspin 12 centrifuge (BioSan, Riga, Latvija), then applied to SEM stubs, dried at room temperature, and sputtered with gold using an ion-plasma sputtering system (Quorum Q150R S plus; Quorum Technologies Ltd., London, UK). For studies with the transmission electron microscope (TEM), formvar-coated grids (EMS FF200-Cu-50, Electron Microscopy Sciences, Hatfield, PA, USA) were used.

The description of the stomatocyst *Mallomonas doignonii* Bourrelly was carried out in accordance with the rules of the international working group ISWG (International Statospore Working Group) [74].

Conducting research on the territory of the “Orenburgskii” State Nature Reserve and the territory of the “Svetlinskii” Biological Reserve was agreed upon with the Government of the Orenburg Region and the “Directorate of Specially Protected Natural Territories of the Orenburg Region”.

## 3. Results

**Figure 2:**
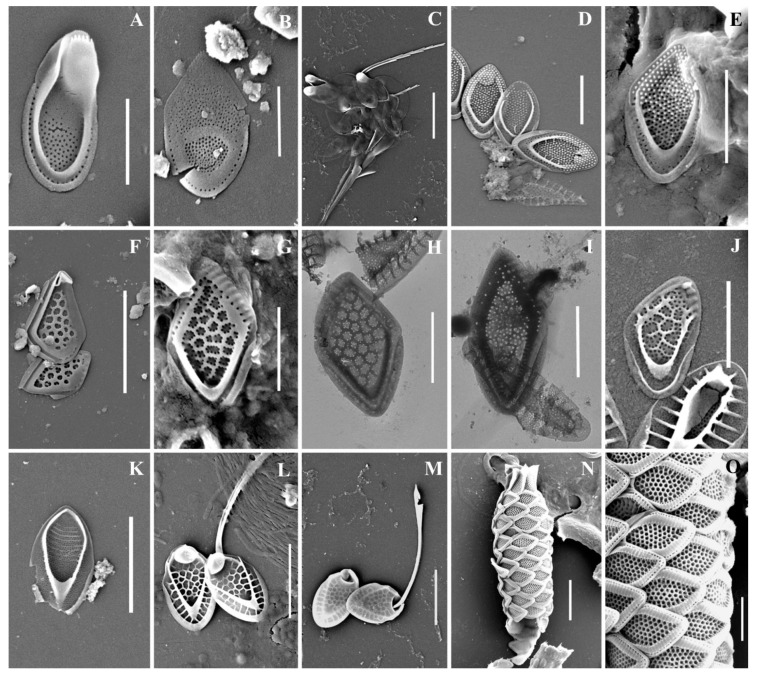
*Mallomonas* taxa from Southern Urals, Russia: (**A**–**C**) *M. akrokomos*, (**A**) apical scale, (**B**) body scale, (**C**) whole cell; (**D**,**E**) *M. annulata*; (**F**–**J**) *M. alata*, (**F**) apical scales, (**G**–**I**) body scales, (**J**) caudal scale; (**K**) *M. costata*; (**L**,**M**) *M. crassisquama*; (**N**,**O**) *M. eoa*. Scale bars: (**C**,**F**,**K**–**N**): 5 µm, (**A**,**B**,**D**,**E**,**G**–**J**,**O**): 2 µm. SEM: (**A**–**G**,**J**–**O**). TEM: (**H**,**I**).

**Figure 3:**
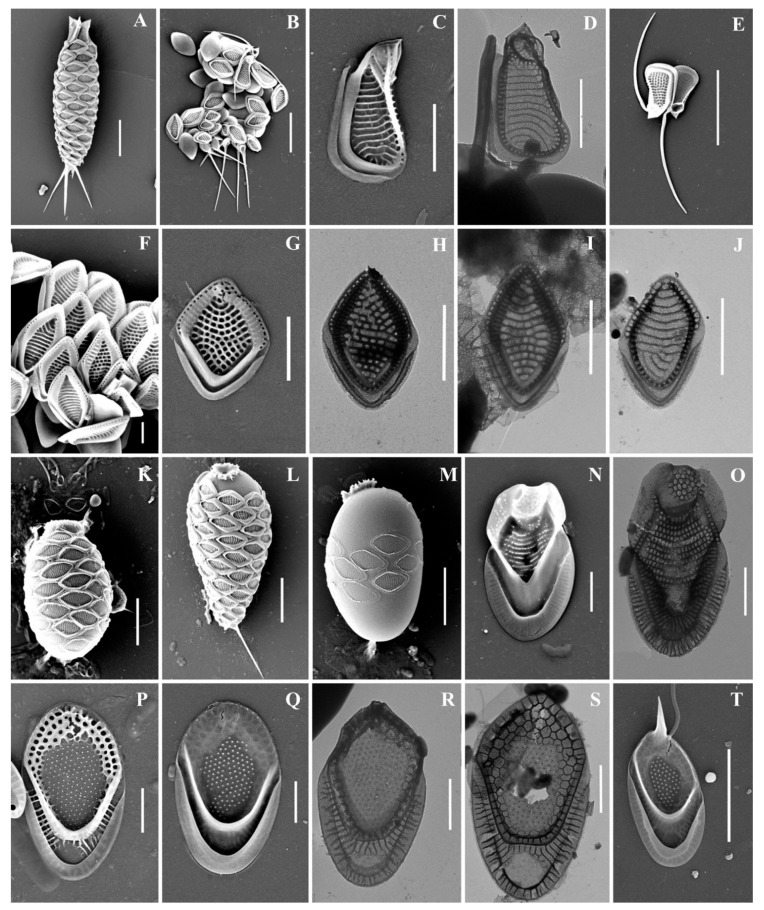
*Mallomonas* taxa from Southern Urals, Russia: (**A**–**M**) *M. doignonii*, (**A**) whole cell, (**B**) different types of scales, (**C**–**E**) collar scales, (**F**–**J**) body scales, (**K**–**M**) stomatocyst 7 Ignatenko et Yatsenko-Stepanova; (**N**,**O**) *M. lelymene*; (**P**–**T**) *M. insignis*, (**P**–**S**) body scales, (**T**) apical scale. Scale bars: (**A**,**B**,**E**,**K**,**L**): 5 µm, (**C**,**D**,**G**–**J**,**M**–**T**): 2 µm, (**F**): 1 µm. SEM: (**A**–**C**,**E**–**G**,**K**–**N**,**P**,**Q**,**T**); TEM: (**D**,**H**–**J**,**O**,**R**,**S**).

**Figure 4:**
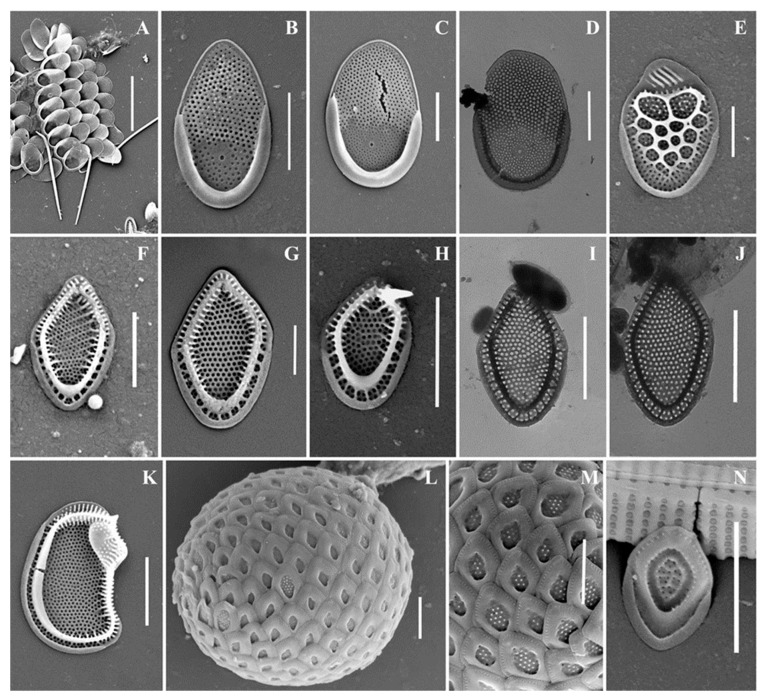
*Mallomonas* taxa from Southern Urals, Russia: (**A**–**D**) *M. matvienkoae*, (**A**) scales with bristles, (**B**–**D**) body scales; (**E**) *M. multiunca*; (**F**–**K**) *M. phasma*, (**F**,**G**,**I**,**J**) body scales, (**K**) collar scale, (**H**) rear scale, one of the anterior submarginal ribs terminates in a short spine; (**L**,**M**) *M. pillula* f. *exannulata*; (**N**) *M. pillula* f. *valdiviana*. Scale bars: (**A**): 10 µm, (**B**–**D**,**F**,**H**–**N**): 2 µm, (**E**,**G**): 1 µm. SEM: (**A**–**C**,**E**–**H**,**K**–**N**); TEM: (**D**,**I**,**J**).

**Figure 5:**
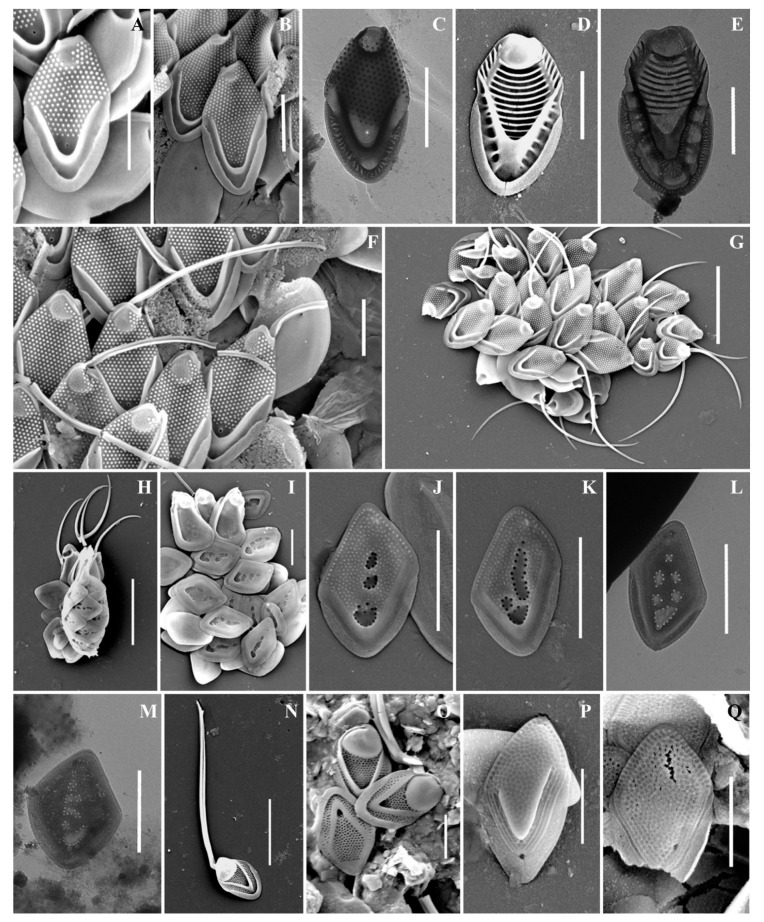
*Mallomonas* taxa from Southern Urals, Russia: (**A**–**C**,**F**,**G**) *M. rasilis*, (**A**–**C**) body scales, (**F**,**G**) scales with bristles; (**D**,**E**) *M. striata*; (**H**–**M**) *M. solea-ferrea* var. *irregularis*, (**H**) whole cell, (**I**) collar and body scales, (**J**–**M**) body scales; (**N**,**O**) *M. tonsurata*; (**P**,**Q**) *Mallomonas* sp. Scale bars: (**G**,**H**,**N**): 5 µm, (**A**–**F**,**J**–**M**,**O**–**Q**): 2 µm. SEM: (**A**,**B**,**D**,**F**–**K**,**N**–**Q**); TEM: (**C**,**E**,**L**,**M**).

**Figure 6:**
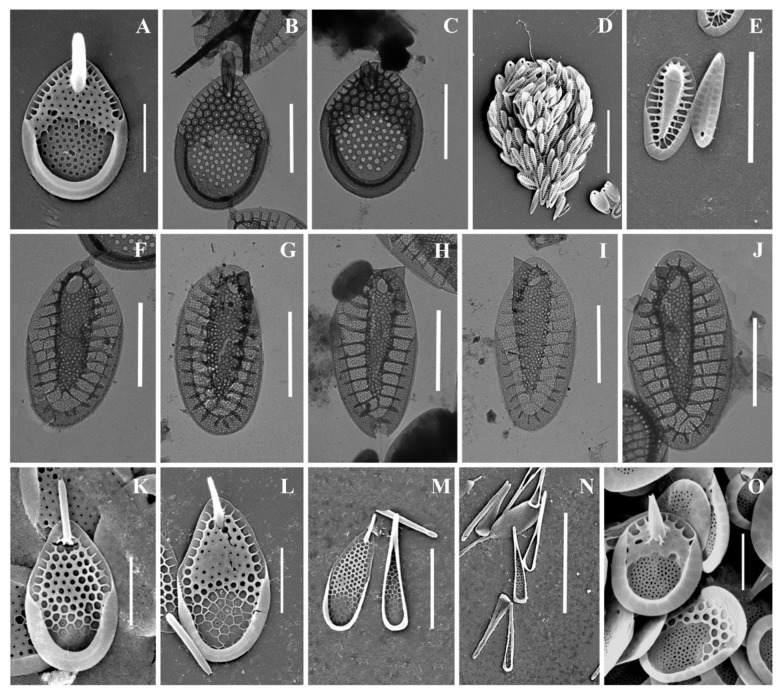
*Synura* taxa from Southern Urals, Russia: (**A**–**C**) *S. curtispina;* (**D**–**J**) *S. petersenii* sensu lato, (**D**) whole cell, (**E**) body and caudal scales, (**F**–**J**) body scales; (**K**–**N**) *S. mollispina*, (**K**,**L**) body scales, (**M**,**N**) caudal scales. (**O**) *S. uvella*. Scale bars: (**D**,**N**): 10 µm, (**E**,**M**): 5 µm, (**A**–**C**,**F**–**L**,**O**): 2 µm. SEM: (**A**,**D**,**E**,**K**–**O**); TEM: (**B**,**C**,**F**–**J**).

**Figure 7:**
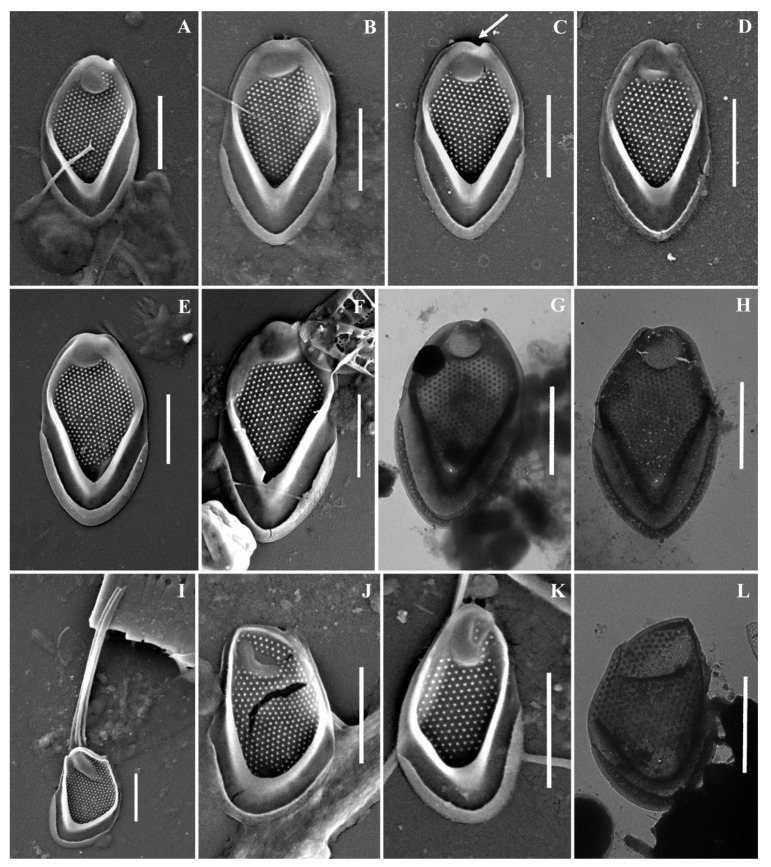
*Mallomonas baturinae* sp. nov.: (**A**–**H**) body scales, (**I**–**L**) apical scales. The arrow marks the notch at the distal end of the scale. Scale bars: 2 µm. SEM: (**A**–**F**,**I**–**K**); TEM: (**G**,**H**,**L**).

As a result of the studies, 24 taxa of silica-scaled chrysophytes were identified (Figure 2, Figure 3, Figure 4, Figure 5, Figure 6 and Figure 7 and Table 2). The representatives of the genus *Mallomonas* Perty were the most numerous (20 taxa), while four taxa belonged to the genus *Synura* Ehrenberg. Twelve taxa were recorded for the first time in the territory of the Southern Urals, including two new species for Russia. One taxon, of the genus *Mallomonas*, is a species new to science. Its description is given below. We could not identify one more taxon of the genus *Mallomonas* to the species level. Presumably, this will also be a new species for science, but additional studies are needed to describe it.

Description of the new species.

**Description**:

Stramenopiles Patterson

Chrysophyceae Pascher

Synurales Andersen

Mallomonadaceae Diesing

*Mallomonas* Perty

***Mallomonas baturinae* sp. nov.** Ignatenko, Gusev & Yatsenko-Stepanova (Figure 7A–L).

Cell dimensions unknown. The body scales are oval, often slightly asymmetrical, 4.6–6.2 × 2.5–3.3 μm, tripartite, with a dome, a V-rib, anterior submarginal ribs, and posterior rim (Figure 7A–H). The anterior submarginal rib and the anterior flange on the right side of the scale form a protrusion that extends further than the central part of the distal end. As a result, at the distal margin there is a notch at the right side of the scale (Figure 7C,D). The dome is circular, occasionally oval, smooth, and surrounded by the arms of the anterior submarginal ribs or joined with them. The shield has densely and regularly spaced papillae. A distinct, rimmed base-plate pore is situated in the proximal area of the shield at the base of the V-rib (Figure 7A,E). The V-rib is conspicuous with an acute angle, and slightly hooded. The distal ends of the arms of the V-rib become continuous with the anterior submarginal ribs. The anterior submarginal ribs are well-developed, wide, and raised above the shield. Anterior flanges are narrow and smooth. The posterior flange is smooth. The posterior rim is narrow and smooth. Apical scales are smaller than the body scales, asymmetrical with an elongated dome, 4.1–4.3 × 2.6–2.9 μm (Figure 7I–L). The bristle is 7.6 μm long, slightly curved, with longitudinal ribs and a tooth at the apex (Figure 7I). Cysts were not observed.

**Holotype specimen:** Portion of a single gathering of cells on SEM stub number 53_I_1 deposited at the Herbarium of the Steppe Institute of the Ural Branch of the Russian Academy of Sciences, Orenburg (ORIS). Material is from the Lake Nezametnoe sampled by M.E. Ignatenko on 29 April 2022. Figure 7D is a representative scale from the type specimen.

**Type locality:** RUSSIA. Orenburg Region: Lake Nezametnoe, Ashchisai steppe, “Orenburgskii” State Nature Reserve, 51°01′20.7″ N, 61°13′29.9″ E, 29 April 2022.

**Etymology:** The species is named in honor of Vera Nikolaevna Baturina (1927–2007) for her contribution to the study of the algal flora of the Ural.

**Distribution and habitat:** This species was found at the type locality and Lake Liman (51°02′19″ N 60°47′09″ E). *Mallomonas baturinae* was found at pH ranged from 6.5 to 8.6 and temperature 11.8–14.8 °C (Table 1 and Table 2).

During the research, a cyst of *Mallomonas doignonii* was also discovered for the first time. We provide a formal description below.

**Stomatocyst 7** Ignatenko & Yatsenko-Stepanova, this paper (Figure 3K–M).

**SEM description:** The stomatocyst is oval (13.5–13.7 × 8.8–9.3 μm) with an unornamented surface. The collar is obliquely conical; the basal diameter of the collar is 2.6–2.8 µm. The collar may be somewhat displaced relative to the central axis of the stomatocyst. The edge of the collar consists of a series of irregularly shaped outgrowths, similar to tentacles. The pore is 0.54 µm in diameter.

**Locality:** RUSSIA, Orenburg region, “Orenburgskii” State Nature Reserve, Lake Nezametnoe, 30 April 2022; *leg. M. Ignatenko*, *T. Yatsenko-Stepanova*.

**Picture-file number:** 9_2, 29, 100.

**Number of observed specimens:** 3.

**Biological affinity:** *Mallomonas doignonii*.

We also found two species of the genus *Mallomonas* new to the flora of Russia: *M. phasma* and *M. solea-ferrea* var. *irregularis*. Both taxa are very rare; these discoveries are the third in the world for these species. Therefore, we provide their extended description and illustrate in detail.

***Mallomonas phasma***(Figure 4F–K). Three types of scales were found: collar scales (Figure 4K), body scales (Figure 4F,G,I,J), and rear scales (Figure 4H). The collar scale is asymmetrical with strongly convex dorsal margin, and measures 4.4 × 2.8 µm. The anterior submarginal rib of the collar scale on the dorsal side is widened, ending in a short spine on the edge from the side of the dome. The dome is wide, covered with ribs and papillae. The base-plate pores are evenly spaced on the shield. The posterior and anterior flanges with the base-plate pores and struts extending from the submarginal ribs both to the edges and to the shield. Body scales are rhombic, measuring 3.1–3.8 × 2.0–2.5 µm, without dome, and with well-developed anterior submarginal ribs. The anterior submarginal ribs have struts extending towards the anterior flanges and shield. The shield is covered with densely and evenly spaced base-plate pores. Sometimes there are thickenings of the secondary siliceous layer, extending from the struts and forming a network structure. The posterior flange has base-plate pores. The rear scale (2.8 × 1.9 µm) is slightly asymmetrical, characterized by the presence of a small spine formed by the continuation of the anterior submarginal rib.

***Mallomonas solea-ferrea* var. *irregularis*** (Figure 5H–M). Cells are ovoid (6.8–10.5 × 3.5–5.2 µm). Bristles are smooth and slightly curved. Collar scales are trapezoid in shape with a well-developed dome, covered with small papillae, and measure 3.0–3.3 × 1.8–1.9 µm. Posterior scales measure 1.4–2.1 × 1.0–1.3 µm. Body scales are rhombic and without dome, measuring 2.9–3.1 × 1.8–2.2 µm. The anterior submarginal ribs on the body scales are not pronounced. The shield is covered with longitudinal rows of small papillae. On the surface of the shield there are irregularly shaped depressions containing pores, most often located along the edges of the depressions.

Since the chrysophytes of the studied region had been hitherto virtually unstudied, and the water bodies of the steppe zone as a whole had been poorly studied, we present a description of new findings of the class Chrysophyceae for the flora of the region with a discussion of their distribution.

***Mallomonas alata*** (Figure 2F–J) is widespread, eurythermal, and indifferent to water acidity (pH) species [4,47]. It has been registered in North and South America, Great Britain, Europe, Asia, and Australia [4]. In the territory of Russia, it was found in water bodies of St. Petersburg and Leningradskaya Oblast [15], Nizhegorodskaya Oblast [28,49], Neneczkii Autonomous Okrug [20], Yamalo-Neneczkii Autonomous Okrug [19], the Republic of Komi [20,35], Krasnoyarskii Krai [17], and the Republic of Buryatia [32].

***Mallomonas annulata*** (Figure 2D,E) is a widespread species recorded in North and South America, Great Britain, Europe, Asia, and Australia [4]. In the territory of Russia, the species was recorded in the water bodies of St. Petersburg and Leningradskaya Oblast [11,15,27], Yamalo-Neneczkii Autonomous Okrug [19], and the Republic of Sakha (Yakutia) [34].

***Mallomonas costata*** (Figure 2K) is a widespread species known in North America, Great Britain, Europe, Asia, and Australia [4]. In the territory of Russia, it was recorded in the water bodies of St. Petersburg, Leningradskaya Oblast [15], Nizhegorodskaya Oblast [28,49], Neneczkii Autonomous Okrug [20], the Republic of Komi [20,35], Krasnoyarskii Krai [45], and the Republics of Buryatia [31,32] and Sakha (Yakutia) [33].

***Mallomonas doignonii*** (Figure 3A–M) was recorded in the middle latitudes of the Northern Hemisphere: North America, European countries, and Great Britain [4]. To date, the only known discovery of *M. doignonii* has been in Russia, at the mouth of the River Barguzin, Republic of Buryatia [32]. We present here the second discovery of this species in the country.

***Mallomonas eoa*** (Figure 2N,O) is a widespread species known in North and South America, Great Britain, Europe, Asia, and Australia [4]. In the territory of Russia, *M. eoa* was previously found in the water bodies of the Republic of Karelia [11], St. Petersburg and Leningradskaya Oblast [15], Yaroslavskaya Oblast, Nizhegorodskaya Oblast [12,13], the Republics of Buryatia [32] and Sakha (Yakutia) [18,34], Bolshezemelskaya tundra [20], Yamalo-Neneczkii Autonomous Okrug [19], and reservoirs of the Volga basin [13].

***Mallomonas insignis*** (Figure 3P–T) is a widespread species, recorded in North America, Great Britain, Europe, Asia, and Australia [4]. On the territory of Russia, *M. insignis* was found in water bodies of the Yamalo-Neneczkii Autonomous Okrug [19], Yaroslavskaya Oblast [13], Nizhegorodskaya Oblast [28], Permskiy Krai [13], Krasnoyarskii Krai [17], and the Republic of Buryatia [31,32].

***Mallomonas lelymene*** (Figure 3N,O) is a widespread species known in North and South America, Great Britain, Europe, and Asia [4]. Discoveries of *M. lelymene* in Russia have not been numerous. Previously, the species was identified in small water bodies in the Neneczkii Autonomous Okrug, Arkhangelskaya Oblast [20], Vladimirskaya Oblast [21], and the Republic of Sakha (Yakutia) [34].

***Mallomonas pillula* f. *exannulata*** (Figure 4L,M) is known in North America and Europe [4,5]. In the territory of Russia, this species was found in two localities: the Republic of Komi [36] and the Nizhegorodskaya Oblast [28].

***Mallomonas pillula* f. *valdiviana*** (Figure 4N) has been recorded in North and South America, Europe, and Asia [4,47]. In Russia, *M. pillula* f. *valdiviana* was found in the Republics of Komi [20,36] and Sakha (Yakutia) [34].

***Mallomonas* sp.** (Figure 5P,Q). The body scales are rhomboidal and slightly asymmetrical, measuring 3.3–3.6 × 2.2–2.4 µm. The shield is patterned with a reticulated meshwork and has scattered papillae. The proximal part of the shield is raised above the posterior submarginal rib. The anterior flange is narrow with struts. The posterior submarginal rib area consists of several (usually three) fine ribs on each side. The posterior flange is smooth, and the posterior border is wide.

***Synura curtispina*** (Figure 6A–C) is a widespread species known in North and South America, Europe, Asia, Africa, and Australia [4]. In the territory of Russia, *S. curtispina* was recorded in water bodies of St. Petersburg and Leningradskaya Oblast [15,26], Nizhegorodskaya Oblast [49], Neneczkii Autonomous Okrug [20], Republic of Komi [20,35], Yamalo-Neneczkii Autonomous Okrug [17], and the Republic of Sakha (Yakutia) [18,34]. Like other species of the genus, the *S. curtispina* morphotype forms several clades on the phylogenetic tree and requires further study [75,76].

## 4. Discussion

Previously, 15 taxa of *Mallomonas* and 13 taxa of *Synura* were recorded in diverse water bodies in the montane-forest zone of the eastern foothills of the Southern Urals and the steppe zone of the Southern Urals using electron microscopy [14,38,50,51,52,53,54,68]. The results obtained in this study made it possible to supplement this list with 13 new taxa, one of which is described as new to science and two (*M. phasma*, *M. solea-ferrea* var. *irregularis*) are the first records in Russia.

Newly described *Mallomonas baturinae* should be placed in the section *Papillosae* based on the presence of papillae on the shield and the presence of the dome on all types of scales. The main distinctive features of the newly described species are the notch at the right side of the scale on the distal margin, which is formed by the protrusion of anterior flange and submarginal rib, the wide and raised anterior submarginal ribs, and the structure of apical scales with an elongated dome. Within this large section, *M. baturinae* is most similar to *M. paxillata* (D.E. Bradley) L.S. Péterfi & Momeu. Scales of both species have a similar size range and a protrusion formed by an anterior flange and submarginal rib adjacent to the dome from one side. However, this protrusion forms a prominent and sharp tooth on the scales of *M. paxillata* [4,75]. The scales of *M. baturinae* only have a rounded protrusion on the right side, which forms a characteristic notch at the distal margin behind the dome. The second important difference between the two species is the structure of the anterior flanges and submarginal ribs. The scales of *M. paxillata* do not have clearly separated anterior submarginal ribs; in their place, there are elevations of the shield covered with papillae. The anterior flanges on *M. paxillata* scales are also covered with papillae. The scales of *M. baturinae* have clearly defined anterior submarginal ribs, which are wide, devoid of papillae, raised high above the shield, and surround the dome or are joined with it. Anterior flanges are narrow and smooth on *M. baturinae* scales. *M. baturinae* is somewhat similar to *M. calceolus* D.E. Bradley [76], *M. kalinae* Rezácova [77], and *M. furtiva* Gusev, Certnerová, Škaloudová & Škaloud [78] in having anterior submarginal ribs and smooth anterior flanges. However, the body scales of *M. baturinae* have the notch on the distal margin and are larger (4.6–6.2 × 2.5–3.3 μm) than those of *M. calceolus* (3–4 × 1–2 μm), *M. kalinae* (3.7–3.9 × 1.7–2.0 μm), and *M. furtiva* (3.6–4.3 × 2.2–2.5 μm). The scales of *M. calceolus* are also distinguished from *M. baturinae* by a lower density of papillae on the shield and much less-developed anterior submarginal ribs. *M. kalinae* and *M. furtiva* are distinguished from *M. baturinae* by less-developed anterior submarginal ribs on the scales and by bristle ultrastructure. *M. kalinae* has smooth pointed bristles and *M. furtiva* has serrated bristles. In both species, domes are shifted towards the distal margin of scales, while on *M. baturinae* scales, the dome is usually surrounded by the wide anterior submarginal ribs and slightly recessed from the distal margin. The scale, which can be attributed as *M. baturinae*, was found in Ob River under the epithet *M. kalinae* ([19], Figure 5I).

Among the taxa found in this study, about half belong to cosmopolitan (*M. akrokomos*, *M. matvienkoae*, *M. rasilis*, *M. tonsurata*) or widespread species (*M. costata*, *M. crassisquama*, *M. striata*, *Synura curtispina*, *S. petersenii*, *S. uvella*) [79]. At the same time, rare species were found: *M. phasma* and *M. solea-ferrea* var. *irregularis*. To date, only two records of *M. phasma* are known in Europe [73,80]. *M. phasma* was first described in 1960 in shallow peat bogs in the southeast of England [80]. Later, Němcová et al. [73] reported this species in four water bodies of various types in southwestern France. Previously, it was considered that *M. phasma* was endemic to Europe [73], but our discovery in the Asian part of Russia expands the range of the species and suggests the possibility of finding *M. phasma* in other regions of the world. Based on the example of *M. phasma*, we consider it necessary to note that the classification of rare species as endemic is often approximate, since, perhaps, their detection only in one region is a consequence of insufficient knowledge. *M. solea-ferrea* var. *irregularis* was first described in 2013 from water bodies of the Czech Republic [81], and for a long time there was no information about its records in other localities. The second discovery of *M. solea-ferrea* var. *irregularis* was registered in 2022 far outside of Europe. The species was recorded in Indonesia, in the high-mountain swamp reservoir of the island of New Guinea [66]. In the present study, we report the third record of *M. solea-ferrea* var. *irregularis*, which indicates a fairly wide range of species distribution. The specimens of *M. solea-ferrea* var. *irregularis* from Southern Urals differ from Czech populations only in their slightly smaller cell sizes (6.8–10.5 × 3.5–5.2 µm versus 9.0–11.8 × 4.0–5.9 µm).

The variability in the ultrastructural organization of the scales and differences in the morphology of the bristles of the morphotypes of *M. rasilis* that were found in the Southern Urals should be noted separately. According to the original description, *M. rasilis* has scales with a dome without papillae and unilaterally serrated bristles [82]. At the same time, scales with a dome partially covered with papillae and with smooth bristles were reported from South Korea [83]. The specimens found in Vietnam were distinguished by the presence of smooth bristles and a dome completely covered with papillae [78]. Scales of *M. rasilis* with a dome completely covered with papillae have also been reported from Madagascar [84], the Czech Republic [62], and Hungary [85]. In our study, two morphotypes of *M. rasilis* were found. One of them had smooth bristles and scales with a dome partially covered with papillae (Figure 5G). The second also had scales with a dome partially covered with papillae, but the bristles were unilaterally serrated (Figure 5F). The size of the scales of the second morphotype was larger than the size indicated in the description of *M. rasilis* (4.1–5.5 × 2.5–2.9 µm versus 3.8–4.1 × 2.1–2.5 µm). In addition, in the case of the second morphotype, along with the typical ornamentation of the shield, there were many scales with the shield completely covered with papillae, including the area of the pore at the base of the V-rib (Figure 5B). Morphological variability, as a response to changes in environmental factors, has been noted in a number of papers. For example, Siver and Skogstad [86] found a clear relationship between water temperature and bristle morphology in *Mallomonas crassisquama*. Using the example of *M. tonsurata*, *M. kalinae*, *Synura petersenii*, *S. curtispina*, a tendency was noted to reduce the size of the scales and the length of the bristles, as well as to change the shape of the scales in response to an increase in temperature of cultivation [87,88,89]. A change in the shape of the scales of *S. echinulata* Korshikov was revealed under the combined action of illumination and temperature, as well as in the pH gradient of the environment [90]. 

We were unable to correlate the morphological variability of scales and bristles of *M. rasilis* that we identified with the ecological adaptation of the species to environmental conditions, since in one sample (Lake Zhurmankol, May 2020) we simultaneously recorded cells covered with scales with smooth bristles, and cells covered with scales with serrated bristles. This observation suggests that the differences in the morphology of the setae of *M. rasilis* in this case are not an ecological adaptation but represent the presence of different morphotypes of this species in the sample. A large number of morphological variations within the morphotype of *M. rasilis* indicates the need for molecular studies, which may confirm the significant polymorphism of the species, or allow the species to be divided into separate taxa.

During the study, a few *Mallomonas* scales were found that we could not identify to the species level. *Mallomonas* sp. (Figure 5P,Q) can be assigned to the *Torquatae* section. According to the scale ultrastructure, *Mallomonas* sp. is similar to *M. favosa* K.H. Nicholls. *M. favosa* f. *favosa* is considered a cosmopolitan species [79]. Initially, *M. favosa* was described in Canada [91] and was later found in other parts of the world [4,67,92,93]. The main difference between *M. favosa* and our discovery is the structure of the posterior submarginal rib. *M. favosa* has the well-delimited posterior submarginal rib, while *Mallomonas* sp. has three or more fine ribs in this area on each arm. We found only two scales of *Mallomonas* sp., which was not enough to describe a new species, and this organism requires additional studies.

During studies of the flora of silica-scaled chrysophytes in the water bodies of the steppe zone of the Southern Urals, several interesting stomatocysts were found [68,69,70]. Stomatocysts are the endogenous siliceous resting stage of the chrysophyte life cycle. Stomatocysts are formed in response to endogenous or exogenous signals, ensuring the preservation of the chrysophyte population [94]. Currently, about 2000 morphotypes of stomatocysts have been described from different regions of the world [95], and only a small number (10–15%) of them are correlated with the vegetative stage [96,97,98]. Stomatocysts, along with other siliceous structures such as scales and bristles, are widely used in paleolimnological studies [95,96]. Unlike scales and bristles, which are preserved for a short geological period, stomatocysts are more heavily silicified and are present in older sediments [1,99]. However, the lack of relationship between most currently known stomatocyst morphotypes and their vegetative stages limits the use of stomatocysts in studying the evolutionary history of chrysophytes [96]. In view of the above, determining the biological identity of stomatocysts (correlation with the vegetative stage) is undoubtedly relevant.

During the study, we discovered and described stomatocyst 7 Ignatenko & Yatsenko-Stepanova (Figure 3K–M); we reliably established that it belongs to *M. doignonii*. This morphotype has a significant similarity to stomatocyst 17, Pang & Wang, 2013, produced by another representative of the *Torquatae* section–*M. eoa* [100,101]. Despite the existing postulate about the species specificity of chrysophycean stomatocysts [96], a high similarity of stomatocysts was also noted among representatives of the genus *Synura*. In particular, it was shown that, due to the morphological simplicity, *Synura* cysts can be almost identical to each other, as well as to stomatocysts of other golden algae [102]. The main difference between the stomatocysts of *M. doignonii* and *M. eoa* is the asymmetry of the collar of the cyst of *M. doignonii*; however, the small number of observed specimens (n = 3) does not allow us to reliably judge the diagnostic significance of this feature; it therefore requires further observations. The high degree of morphological similarity of the stomatocysts of *M. eoa* and *M. doignonii* can cause identification errors and, as a result, distort the information of the range and biogeography of these species.

The genus *Synura* in our study was represented by a smaller number of taxa than *Mallomonas*. We found four species from sections *Synura* and *Petersenianae*. *S. curtispina* is noted as a new species for the flora of the Southern Urals.

Representatives of the *S. petersenii* sensu lato species complex were also found in our study. The *S. petersenii* s.l. complex includes 17 genetically characterized species, as well as a number of taxa belonging to the section *Petersenianae* that are still unexplored in detail to date; their phylogenetic position is still unknown [5,103]. Due to the high morphological similarity, the identification of the species of the *S. petersenii* s.l. complex is difficult. For the correct identification of these species, it is necessary to involve molecular methods. In our study, we were unable to identify the detected scales down to the species level without molecular data, and we identified them as *S. petersenii* s.l. At the same time, we consider it necessary to publish their images to demonstrate the diversity of silica-scaled chrysophytes in the waterbodies of the steppe zone of the Southern Urals.

## 5. Conclusions

The study of only a small number of steppe water bodies made it possible to discover a new species for science: *Mallomonas baturinae*. Seasonal study of algae made it possible to identify and describe the *M. doignonii* stomatocyst. During our studies, new and very interesting findings were revealed in terms of the biogeography of algae. In particular, steppe water bodies became the third known habitat for two very rare taxa of the genus *Mallomonas*: *M. phasma* and *M. solea-ferrea* var. *irregularis*, which were also new to the flora of Russia. Additionally, among the species that were rare among the flora of Russia were *M. doignonii*, *M. pillula* f. *exannulata*, and *M. pillula* f. *valdiviana*. The diversity of *M. rasilis* morphotypes was revealed. The significant quantity of new data obtained in the study of steppe water bodies of protected natural areas indicates the need for further study of the region.

## Figures and Tables

**Table 1:** List of the sampling sites with environmental variables (T–temperature, S–salinity).

Locality Name	Date	Location	Coordinates	T °C	pH	S, ‰
Lake Nezametnoe	29 April 2022	Ashchisai steppe, “Orenburgskii” State Nature Reserve	51°01′20.7″ N 61°13′29.9″ E	11.8	6.48	0.07
Lake Zhurmankol	13 May 2020	Ashchisai steppe, “Orenburgskii” State Nature Reserve	50°58′31.0″ N 61°09′20.1″ E	19.0	6.82	0.28
	30 April 2022			11.4	6.21	0.81
	30 May 2022			14.5	6.79	0.35
	3 May 2023			15.0	7.11	0.08
Pond Prikordonnyi	13 May 2020	Ashchisai steppe, “Orenburgskii” State Nature Reserve	50°57′43.5″ N 61°12′56.5″ E	17.1	7.03	0.1
	29 April 2022			12.0	6.72	0.08
	30 May 2022			14.6	8.07	0.1
Lake Liman	31 May 2022	“Svetlinskii” Biological Reserve	51°02′19″ N 60°47′09″ E	20.6	6.91	0.06
	3 May 2023			14.8	8.6	0.03

**Table 2:** List of silica-scaled chrysophytes (Chrysophyceae, Synurales) found in water bodies of natural protected areas of the steppe zone of the Southern Urals, Russia (bold type indicates new taxa for the flora of the Southern Urals; “+”/“–” presence/absence of the species in the studied water body).

Taxon	Lake Nezametnoe	Lake Zhurmankol				Pond Prikordonny			Lake Liman	
	Date of Sampling									
	April 2022	May 2020	April 2022	May 2022	May 2023	May 2020	April 2022	May 2022	May 2022	May 2023
*Mallomonas akrokomos* Ruttner (Figure 2A–C)	–	–	–	–	+	–	–	+	+	+
***M. alata* Asmund, Cronberg & Dürrschmidt** (Figure 2F–J)	+	–	–	–	+	–	–	–	–	+
***M. annulata* (D.E. Bradley) K. Harris** (Figure 2D,E)	+	–	–	–	+	–	–	–	–	–
***M. baturinae* sp. nov.** (Figure 7A–L)	+	–	–	–	–	–	–	–	–	+
***M. costata* Dürrschmidt** (Figure 2K)	–	–	–	–	–	–	–	+	–	–
*M. crassisquama* (Asmund) Fott (Figure 2L,M)	–	–	–	–	–	–	+	+	–	–
***M. doignonii* Bourrelly** (Figure 3A–M)	+	–	–	–	–	–	–	–	+	+
***M. eoa* E. Takahashi** (Figure 2N,O)	–	–	–	+	–	–	–	–	–	–
***M. insignis* Penard** (Figure 3P–T)	+	–	–	+	–	–	–	–	–	–
***M. lelymene* K. Harris & D.E. Bradley** (Figure 3N,O)	+	–	–	–	–	–	–	–	+	+
*M. matvienkoae* B. Asmund & Kristiansen (Figure 4A–D)	+	+	–	–	–	–	–	–	+	+
*M. multiunca* Asmund (Figure 4E)	+	–	–	–	+	–	–	–	+	+
***M. phasma* K. Harris & D.E. Bradley** (Figure 4F–K)	+	–	–	–	–	–	–	–	–	+
***M. pillula* f. *exannulata* K. Harris** (Figure 4L,M)	–	–	–	–	–	–	–	–	–	+
***M. pillula* f. *valdiviana* Dürrschmidt** (Figure 4N)	–	–	–	–	–	–	–	–	–	+
*M. rasilis* Dürrschmidt (Figure 5A–C,F,G)	+	+	–	–	+	–	–	–	+	+
***M. solea-ferrea* var. *irregularis* Nemcová, Kreidlová, Pusztai & Neustupa** (Figure 5H–M)	+	–	–	–	–	–	–	–	+	+
*M. striata* Asmund (Figure 5D,E)	+	+	–	–	–	–	+	–	+	+
*M. tonsurata* Teiling (Figure 5N,O)	–	–	–	–	–	–	+	–	–	–
*Mallomonas* sp. (Figure 5P,Q)	+	+	–	–	–	–	–	–	–	+
***Synura curtispina* (J.B. Petersen & J.B. Hansen) Asmund** (Figure 6A–C)	+	+	–	+	–	+	–	+	–	–
*S. mollispina* (J.B. Petersen & J.B. Hansen) Péterfi & Momeu (Figure 6K–N)	–	–	–	+	–	–	–	–	–	–
*S. petersenii* Korshikov sensu lato (Figure 6D–J)	+	+	+	+	+	+	+	+	+	+
*S. uvella* Ehrenberg (Figure 6O)	–	+	–	+	–	–	+	+	+	+

## Data Availability

Data are contained within the article.
